# Ultrasound Assisted Extraction Approach to Test the Effect of Elastic Rubber Nettings on the N-Nitrosamines Content of Ham Meat Samples

**DOI:** 10.3390/foods10112564

**Published:** 2021-10-24

**Authors:** Claudia Giménez-Campillo, Marta Pastor-Belda, Natalia Campillo, Juan de Dios Hernández, Isidro Guillén, Pascuala Vizcaíno, Ignacio López-García, Manuel Hernández-Córdoba, Natalia Arroyo-Manzanares, Pilar Viñas

**Affiliations:** 1 Department of Analytical Chemistry, Faculty of Chemistry, Regional Campus of International Excellence “Campus Mare Nostrum”, University of Murcia, E-30100 Murcia, Spain; claudia.gimenez@um.es (C.G.-C.); marta.pastor@um.es (M.P.-B.); ncampi@um.es (N.C.); ilgarcia@um.es (I.L.-G.); hcordoba@um.es (M.H.-C.); 2 Productos del Sur S.A. (Prosur) Av. Francisco Salzillo, P/27-2, San Ginés, 30169 Murcia, Spain; juandedios@prosur.es (J.d.D.H.); isidro@prosur.es (I.G.); pvizcaino@um.es (P.V.)

**Keywords:** nitrosamines, elastic rubber nettings, ham, dispersive liquid–liquid microextraction, gas chromatography-mass spectrometry

## Abstract

Nitrosamines (NAs), which are catalogued as carcinogenic compounds, may be present in meat products due to the conversion of nitrites and as result of migration from elastic rubber nettings used. A method based on ultrasonic assisted extraction coupled with dispersive liquid–liquid microextraction as sample treatment and gas chromatography-mass spectrometry as separation and detection technique was proposed for the determination of twelve NAs in cooked ham samples. The method was validated by evaluating linearity (0.5–1000 ng g^−1^), matrix effect, sensitivity (detection limits were between 0.15 and 1.4 ng g^−1^) and precision, which was below 12%. Five NAs were found in the samples with levels ranging from not quantifiable to 40 ng g^−1^. The effect of the elastic rubber nettings on the nitrosamine content of meat was evaluated by comparing the levels found in products made with several plastics or thread in the presence of additives.

## 1. Introduction

Food security is attracting increasing attention worldwide, especially in regard to the meat industry, which offers numerous products whose safety must be monitored for health preservation [1,2]. The use of nitrite anion as a preservative agent in meat processing at high temperatures can act as precursor in the formation of nitrosamines (NAs) due to its reaction with amines or amides [3,4]. The use of nitrosating agents (nitrites and nitrates) is severely controlled in the meat industry, where they are added to achieve a specific taste, colour and texture, as well as to increase the shelf life of products and prevent rancidity during storage [5]. The nitrite content in meat is one of the most important factors influencing the formation of NAs, along with others that include cooking procedure, time and temperature, existence of precursors, catalysts and inhibitors (ascorbic acid or tocopherols), pre-processing methods, smoking and storage conditions [4].

Another source of NAs in processed meat may be the materials that come into contact with the product such as packaging papers, waxed containers and, especially, elastic rubber nettings [6,7,8,9,10,11]. During the rubber vulcanization process involved in the synthesis of these materials, the accelerators or stabilizers used can originate NAs themselves [12] or amine derivatives (such as dithiocarbamates, thiuram mono- and poly-sulfides, sulfonamides and thioureas), which are present at higher concentrations than the NAs, and which can migrate to the meat [13,14]. The migrated amines can react with the nitrite present in meat to form more NAs during processing or cooking [6,7,8]. It has been demonstrated that the concentration of the NAs formed during these processes is higher on the meat surface than inside [6,15].

The first NAs detected in processed meat, N-nitrosodibutylamine (NDBA) and N-nitrosodiethylamine (NDEA), were found to have migrated from rubber nettings [7,15]. Some years later, N-nitrosodibenzylamine (NDBzA) was detected instead of NDBA due to reformulation of the rubber synthesis process [13,15]. Other NAs, such as N-nitrosodimethylamine (NDMA), N-nitrosopiperidine (NPIP), N-nitrosopyrrolidine (NPYR), N-nitrosomorpholine (NMOR) and N-nitrosodiphenylamine (NDPhA), have been detected in rubber chemicals and rubber products, but there is no information concerning the migration of these chemicals to food [6,12]. Although NAs can appear as secondary products in food preparation and processing, they can also be produced in the environment and within the human body itself [2].

Volatile NAs (NDMA, NDEA, NPYR, NMOR, N-nitrosodi-n-propylamine (NDPA), NPIP, NDBA and NDPhA) are classified by the International Agency for Research on Cancer (IARC) as carcinogenic and mutagenic compounds [1,5]. Any legislation, therefore, must strike a balance between the risk of the formation of NAs through the addition of nitrites and the benefits they offer against microbiological contamination [16]. The tolerable level of NA exposure in humans is 5–10 µg kg^−1^ of body weight per day [4], and thus to limit exposure the World Health Organization (WHO) has set the maximum level of total volatile NAs in processed meat at 10 µg kg^−1^ [17]. European Union (EU) legislation permits nitrite and nitrate to be added to meat products up to a maximum concentration of 150 mg kg^−1^ for each additive, while Denmark only permits the use of 60 mg kg^−1^ of nitrites for meat preservation purposes in Danish products [18]. However, further studies are required to determine whether the addition of either level (150 or 60 mg kg^−1^) of nitrite causes an increase in average nitrosamine levels, for which no maximum limits have been established for processed meat products by the EU.

As human exposure to NAs is mainly through the diet, foods and beverages such as beer [1,19], red wine [20], drinking water [19], meat [1,2,3,4,5,21,22,23,24], fish [25], dairy products [26] and vegetables [27] have been widely analysed. Gas chromatography (GC) is the most frequently used technique [2,3,4,5,19,20,22,24,25,26,27,28,29,30,31], with mass spectrometry (MS) usually selected as detector [3,5,19,20,22,24,27,28,29] due to its high selectivity and sensitivity. Liquid chromatography has also been applied [1,21,32,33], in some cases involving a derivatization reaction [30]. Because NAs are generally present at low concentrations and samples tend to have complex matrices, a sample treatment step, based on cleaning and/or pre-concentration, is essential for their analysis [34]. Traditional techniques such as solid-phase extraction (SPE) [3,21,26,27,28,29,31] and liquid–liquid extraction (LLE) [29,35] have been the most frequently selected techniques, along with others such as supercritical fluid extraction (SFE) [4]. Microextraction techniques, which are environmentally friendly and in accordance with the principles of green analytical chemistry, have also been applied [36,37,38,39,40]. In the case of meat analyses, solid-phase microextraction (SPME) [19,20,22,24] and dispersive liquid–liquid microextraction (DLLME) [1,2,5,32,41] are the most widely adopted miniaturized approaches.

This work evaluates the effect of elastic rubber nettings on the contents of twelve N-nitrosamines in ham meat samples. The procedure is based on the combination of ultrasound-assisted extraction (UAE) and DLLME for the extraction and preconcentration of NAs from cooked ham samples, while GC–MS is applied to analyze the extracts. Moreover, the effect of temperature on NA levels in the samples was evaluated.

## 2. Materials and Methods

### 2.1. Reagents

A certified reference material EPA 8270/Appendix IX Nitrosamines with 2000 μg mL^−1^ of N-nitrosodibutylamine (NDBA), N-nitrosodiethylamine (NDEA), N-nitrosodimethylamine (NDMA), N-nitrosodiphenylamine (NDPhA), N-nitrosodi-n-propylamine (NDPA), N-nitrosoethylmethylamine (NEMA), 1-nitrosopiperidine (NPIP) and 1-nitrosopyrrolidine (NPYR) in methanol (MeOH), was obtained from Sigma Aldrich (St. Louis, MO, USA). Individual standards, with purities of 95–98%, of N-nitroso-methylphenylamine (NMPhA), N-nitroso-ethylphenylamine (NEPhA), N-nitrosodiisobutylamine (NDiBA) and N-nitrosodibenzylamine (NDBzA) were provided by Toronto Research Chemicals (Luckenwalde, Germany) and individual solutions were prepared at 1000 μg mL^−1^ in MeOH. All concentrated standard solutions were kept in the freezer at −20 °C. A standard working solution containing the NAs at 10 μg mL^−1^ was prepared every day in MeOH. Organic solvents, including acetone, acetonitrile (ACN) and MeOH of chromatographic quality grade, were purchased from Chem-Lab (Zedelgem, Belgium). Carbon tetrachloride, chlorobenzene, chloroform, 1-dodecanol and 1,1,2,2-tetrachloroethane were used as extractant organic solvents (Sigma-Aldrich, St. Louis, MO, USA). Water from a Milli-Q system (Millipore, Bedford, MA, USA) was employed. Other reagents were sodium chloride, trichloroacetic acid (TCA) and sodium hydroxide (Sigma) and hydrochloric acid (25% m/m) from Riedel-de-Häen (Wunstarfer, Germany). The internal standard (IS) 2-octanone was provided by Sigma-Aldrich.

### 2.2. Instrumentation

A multipurpose sampler (MPS, Gerstel, Mülheim, Germany) able to work in both modes of direct injection and headspace and an 8890 gas chromatograph (Agilent Technologies, Santa Clara, CA, USA) were used. MS detector was an Agilent 5977B quadrupole with an inert ion source. The capillary column (30 m × 0.25 mm I.D., 0.25 µm film thickness) was HP-5MS UI (5% diphenyl–95% dimethylpolysiloxane, Agilent). The helium flow was 1 mL min^−1^ and injection volume 1 µL in splitless mode. The GC oven program was as follows: initial temperature at 50 °C, held for 3 min; increase to 80 °C at 20 °C min^−1^ (2 min); increase to 100 °C at 5 °C min^−1^ (2 min) and final temperature of 280 °C at 35 °C min^−1^ (2.36 min). Under these experimental conditions, the compounds eluted in the 4.01–18.02 min range, these values corresponding to NDMA and NDBzA, respectively. The ion source, transfer line and quadrupole were set at temperatures of 230, 250 and 150 °C, respectively. The MS operated using electron-impact (EI) mode at 70 eV. Selection of experimental conditions was performed using full scan method, while validation and sample analysis were conducted with one target and two or three qualifier ions in selected ion monitoring (SIM) mode. The retention times and the selected ions in SIM mode are shown in Table 1. The MassHunter Workstation Data Acquisition software (Agilent Technologies, Rev.B.08.00) was applied for data processing. Quantification was carried out in the peak area of the extracted ion chromatogram (EIC) of the target ion.

An IKA A11 basic (Wilmington, USA) mixer was used to crush and homogenize the meat samples (25 g) before storage in the freezer at −20 °C until analysis. An UP 200H ultrasonic probe processor (Dr. Hielscher, Teltow, Germany) provided with a titanium sonotrode (7 mm I.D.) and an effective output of 200 W in liquid media was used to extract the NAs from the sample matrices. Two types of centrifuge were used: an EBA 20 (Hettich, Tuttlingen, Germany) operating at 3000 rpm and an MPW-150R (Warsaw, Poland) operating at 6000 rpm at 10 °C. Sample extracts were filtered with 1 mL needle-free Nipro Syringes and nylon filters (25 mm, 0.45 µm) (Agilent Technologies). A TQTECH 2001244 drying oven (Murcia, Spain) with adjustable temperature between 40 and 250 °C was used to heat the samples.

### 2.3. Samples and Analytical Procedure

Seven different meat samples were manufactured and provided by Prosur (Productos del Sur, Murcia, Spain). Each sample was manufactured with a different type of meat and with different additives and plastic coatings. All samples were made of pig meat except for sample 5, which was made of free-range chicken meat. Nitrite ion and a polyphenol-rich extract (NATPRE T-10 HT S) were used as additives. Sample 2 did not contain any type of additive, while the rest of the samples contained NATPRE (samples 1, 5 and 6), ecological NATPRE (sample 3) or nitrite at different concentrations (20 and 150 mg kg^−1^ nitrite in samples 4 and 7, respectively). Samples 2 to 7 were cooked wrapped in three types of plastic coating: prolan M-0 V-6-4 HGB-3 XL (sample 6), prolan V-22 (sample 2–5) and prolan V-66 HGB-45 REG (sample 7), while sample 1, which was coated with a thread netting, was unwrapped before cooking.

The hams contained 90% meat, 1.5% salt, 0.5% phosphate, 8% water and different amounts of sodium nitrite (20 or 150 mg NaNO_2_ kg^−1^) or NATPRE T-10 HT S (20 g kg^−1^). The final weight of each ham was 5 kg. Non-meat ingredients were placed in a vacuum mixer (CATO, Girona, Spain) together with the ground ham and mixed for 1 h. The resulting meat–brine mixture was stuffed in sausage casing made of different elastic rubber nettings and cooked until reaching a core temperature of 68 °C (the maximum oven temperature was 73–75 °C). Hams were chilled to 37 °C within 1.5 h and to 4 °C within 4.5 h. The weight after the chilling process was checked to ensure that all the brine had been absorbed by the meat. The samples were frozen and stored for a period of two months before analysis.

The analytical procedure consisted of weighing 1 g of the homogenized meat sample into a 15 mL Falcon tube and adding 10 mL of water. The mixture was placed in an ice bath and submitted to ultrasounds for 5 min using a probe operating with 0.75 s pulses of 105 μm amplitude for extracting the NAs from the meat. Subsequently, 1 mL of 40% m/v TCA was added to precipitate the proteins, and the mixture was centrifuged at 6000 rpm and 10 °C for 5 min. The resulting supernatant was filtered through 0.45 µm nylon filters, diluted with water up to 10 mL and located in a 15 mL conical bottomed glass tube. For the DLLME step, a mixture of 0.5 mL MeOH (dispersant solvent) with 120 µL chloroform (extractant solvent) was injected quickly into the aqueous solution containing 0.5 g of NaCl (5% m/v concentration). Thus, micro-droplets of chloroform were dispersed in the aqueous phase and were centrifuged at 3000 rpm for 3 min, and a volume of 30 µL of the drop deposited was placed in a 2 mL vial with a 250 µL micro-insert with a polymeric foot. Next, 2 µL of a standard solution of 2-octanone at 3 µg mL^−1^ was added to the CHCl_3_ drop as an internal standard (IS). A 1 µL-volume of the resulting solution was injected automatically into the GC–MS system.

## 3. Results

### 3.1. Optimization of Sample Preparation

The injection mode into the gas chromatograph was the first parameter studied for both direct and headspace modes. For headspace injection, volumes of 5 mL of 10 µg mL^−1^ NA standard solutions prepared in water, 1,1,2,2-tetrachloroethane and 1-dodecanol were submitted to different incubation temperatures (90, 110 and 130 °C) for 30 min. The headspace mode was also assayed with 2.5 g of a homogenized cooked ham sample fortified at 100 µg g^−1^ NAs in the absence and in the presence of 5 mL water or 1-dodecanol and in both cases incubated at 90 °C. Headspace mode provided the best results for the meat sample submitted to an incubation temperature of 110 °C for 30 min and in the absence of solvent. Direct injection mode was tested by injecting 1 µL of methanolic solution of NAs at 10 µg mL^−1^. Comparison of the results obtained with both injection modes showed that the chromatographic peaks were well defined and sensitivity was 2–10 times greater using direct injection, which was chosen.

**Figure 1:**
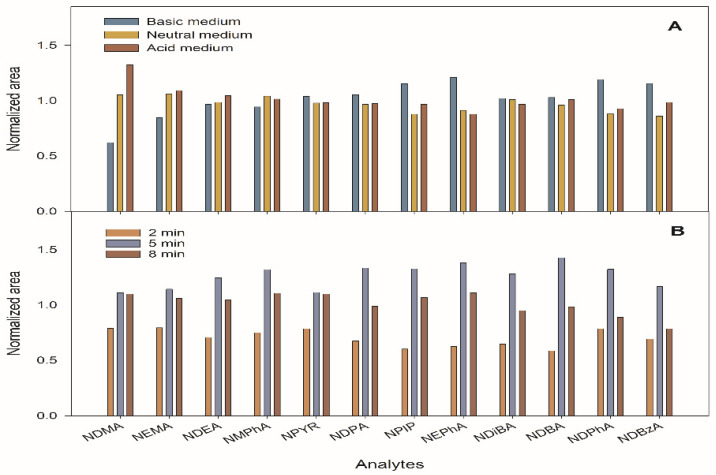
Influence of the nitrosamine (NA) extract medium (**A**) and the duration of ultrasounds treatment carried out by means of a probe (**B**) in the meat samples using ultrasound-assisted extraction (UAE).

The extraction of the analytes from the meat matrix was studied using 0.5 g of a sample fortified at 100 ng g^−1^ to which 10 mL of 0.05 M NaOH, water or 0.05 M HCl were added, and the mixtures were submitted to ultrasounds for 2 min using a directly immersed probe. The meat sample totally dissolved in alkaline medium and partially dissolved in both water and acidic media. As there were no significant differences between the extraction media in this respect, water extraction was selected. Next, 1 mL of 40% m/v TCA was added to the solution to precipitate the meat proteins. After the acidification process with TCA, the need to adjust the pH of aqueous phase was varied in the 1–8 range. The results showed that no such adjustment was necessary because there were no significant differences in the NA extraction efficiency at any value (Figure 1A).

The length of ultrasounds application was also optimized by testing for 2, 5 and 8 min, operating with pulses of 0.75 s or 105 μm amplitude. As shown in Figure 1B, maximum extraction efficiencies were reached for a 5 min application time, which was selected. However, repeatability of the experiments was poor due to sample overheating during the ultrasound probe application and the high volatility of some NAs. For this reason, the UAE step was carried out by maintaining the sample mixture in an ice bath. Subsequently, a centrifugation step was included, and the optimal centrifugation time at 6000 rpm at 10 °C was tested between 2 and 10 min. It was observed that 5 min was sufficient to obtain a clean supernatant phase. The supernatant was filtered using 0.45 µm nylon filters. The liquid phase obtained (approximately 8.5 mL) was diluted up to 10 mL with water and submitted to the DLLME procedure.

The sample mass was optimized by applying the above procedure with masses of 0.25, 0.5, 0.75, 1 and 1.5 g of pig meat sample fortified at 100 ng g^−1^. Analytical signals for each NA proportionally increased with meat mass up to 1 g, above which they remained constant. So, a mass of 1 g of meat was selected. The volume of the extraction solution was also optimized by adding 5, 10 and 12 mL of water to 1 g of sample. Similar results were found using 10 and 12 mL, and thus a 10 mL volume was selected.

NAs were preconcentrated using DLLME [2,42], checking the suitability of chloroform and MeOH as extractant and dispersant solvents, respectively, for the twelve NAs studied. Thus, the experimental conditions related to the volume of the three DLLME phases, as well the salt content in the aqueous phase were those selected in a previous study [42]. The influence of the extractant solvent volume was studied between 100 and 150 µL. Lower extractant volumes were not tested because no sedimented drop was obtained. A volume of 120 µL of chloroform provided the maximum signal for all the NAs and was therefore selected. In summary, the experimental conditions were: 0.5 mL MeOH, 120 µL chloroform and 10 mL of aqueous solution containing 5% m/v NaCl.

### 3.2. Method Validation

In accordance with international guidelines [43], the method was validated based on several criteria: linearity, detection (DL) and quantification (QL) limits, selectivity, recovery studies and precision.

Calibration curves were prepared in the absence and in the presence of the sample matrix (ham and chicken) using 6 concentration levels of between 1 and 1000 ng g^−1^, applying the proposed procedure. The final sedimented drop was fortified with 6 ng of IS (2-octanone, with an intermediate retention time of 8.27 min, was used as IS after checking its absence in the samples) and 1 µL was injected into the GC–MS, thus compensating for any losses that occurred during the GC injection step. Calibration graphs represented the ratio of the NA peak area and the IS peak area vs. the concentration and were adjusted using the internal standard method (R^2^ > 0.99 in all cases). The slopes, with and without matrix, showed significant differences using an ANOVA test because p-values were lower than 0.05 for all the NAs. This matrix effect prevented sample quantification against aqueous standards. However, no significant differences were found for the slopes of the different meat samples (ANOVA test, *p*-values > 0.05), and thus the mean slope value for each NA was used to quantify the samples by matrix-matched calibration.

The linearity range in cooked ham was 1–1000 ng g^−1^ for NMPhA, NDPA, NEPhA and NDBA, 2.5–1000 ng g^−1^ for NPIP, NDiBA and NDPhA and 5–1000 ng g^−1^ for NDMA, NEMA, NDEA, NPYR and NDBzA (Table 2).

The sensitivity of the method was assessed from the DL and QL values, calculating the concentrations providing analytical signals 3 and 10 times those of the noise, respectively (Table 2). QL values ranged between 0.5 and 4.6 ng g^−1^, which corresponded to NDBA and NDBzA, respectively.

The precision of the method was evaluated in terms of repeatability (intraday analysis) and reproducibility (inter-day analysis) studies. The repeatability of the procedure was tested by preparing three equal aliquots of cooked ham fortified at 100 ng g^−1^ for all the analytes on the same day and injecting each one three times (*n* = 9). The same procedure was carried out on three consecutive days (3 samples each day and each sample injected 3 times) in order to evaluate reproducibility of the method (*n* = 21). The relative standard deviation (RSD) values for each NA, appear in Table 2, being lower than 10% and 12% for intra- and inter-day analysis, respectively. The addition of the IS was seen to improve the repeatability of the method by 7–10%, depending on the compound.

The proposed method was compared with published methods dealing with the NA determination in meat by GC–MS (Table 3) and showed the UAE technique using a probe to be more rapid and easier to apply than microwave-assisted extraction (MAE), which also requires more expensive equipment unless a domestic microwave oven is used [2,24,41]. As regards the preconcentration step, even though SPE [3] has been seen to provide high sensitivity, its inherent disadvantages in being a conventional technique (high consumption of organic solvents and long application times) have promoted the use of miniaturized techniques. In this sense, SPME allows the extraction step to be omitted, but with no greater sensitivity in the case of NAs [15,22]. The high cost and low robustness of the fibers, as well as the longer times necessary for the sample preconcentration step compared with DLLME, also need to be considered. Taking all the above into consideration leads us to recommend the UAE-DLLME combination as a very good choice.

### 3.3. Analysis of Meat Samples and Recovery Studies

Portions of the outer meat (in contact with the protective plastic) and portions of the inner meat from seven different samples of cooked meat were analysed in triplicate. The results pointed to no significant differences between the different portions of the samples when a signed rank test was used (*p*-values between 0.056–0.324). Five NAs (NDMA, NDBA, NDPhA, NMPhA and NDBzA) were detected in all the analyzed samples (Table 4), while non-quantifiable levels of NDEA appeared in sample 7. NDMA was found at concentrations between the detection limit and 14 ng g^−1^, the maximum concentration occurring in the chicken meat samples. NDBA appeared in all the samples at concentrations between 22 and 40 ng g^−1^, the highest value corresponding to the sample which contained 150 mg kg^−1^ nitrite as additive. NDPhA and NMPhA were found in all the meat samples in the 1.9–3.7 and 1.4–2.3 ng g^−1^ ranges, respectively, with no significant differences between the different meat samples. NDBzA was only found in two pig-meat samples, the highest value (28 ng g^−1^) corresponding to the sample to which nitrite had been added at 20 mg kg^−1^.

A comparison of the NAs levels here found with others previously reported between 1992 and 2003 revealed a considerable decreasing of concentration. NDEA and NDiBA were detected at 6.9 ng g^−1^ [6] and 33.5 ng g^−1^ [7], respectively, being not found in the samples here studied. A NDMA level of 70 ng g^−1^ was found by Bouma et al. [13], meanwhile the highest concentration now found is 14 ng g^−1^. NDBA and NDBzA were the NAs with higher occurrence. The literature shows NDBA concentrations in the 50–500 ng g^−1^ range [6,8,10,11], whereas levels lower than 40 ng g^−1^ have now been detected. A significant decreased in the contents of NDBzA was found because only two samples contained traces of this NA (at a maximum level of 28 ng g^−1^), and the contents provided in the literature vary between 980–60 ng g^−1^ [7,8,9,10,13]. The decrease of nitrosamine content with the years can be justified due to the reformulation of elastic rubber nettings applying different alternative chemicals used as accelerators to avoid the formation of NAs during meat curing or processing [44].

The one-way ANOVA performed to evaluate the NA content of the meat samples analyzed identified no significant differences between the content of each NA in the different samples: p-values of 0.425, 0.056, 0.137, 0.740 and 0.082 for NDMA, NDBA, NDPhA, NMPhA and NDBzA, respectively. This allowed us to conclude that the NAs contained in the meat samples were not due to the elastic rubber nettings used in the manufacturing processes of the meat products.

The possible influence of heat on NA migration from the rubber netting to the meat was evaluated using the three samples containing the greatest number of compounds at the highest concentrations (Sample 1, 2 and 4). For this study, a mass of 10 g of a sample containing aliquots of different areas from the meat piece, as well as the plastic material of its wrapping, were placed in an oven programmed at different temperatures (60, 80 and 100 °C) for 30 min. Then the ham was cooled, crushed and analyzed by the optimized procedure. The results obtained were similar for all the samples, the levels of NMPhA and NDBA remaining constant despite the differences in temperature, although a very slight decrease in the signal was observed at 100 °C. In the case of NDiBA and NDBzA, the signal increased slightly up to 80 °C, when they both reached their maximum concentrations. The concentration of NDPhA was constant from room temperature to 80 °C but slightly increased when a temperature of 100 °C was applied.

**Figure 2:**
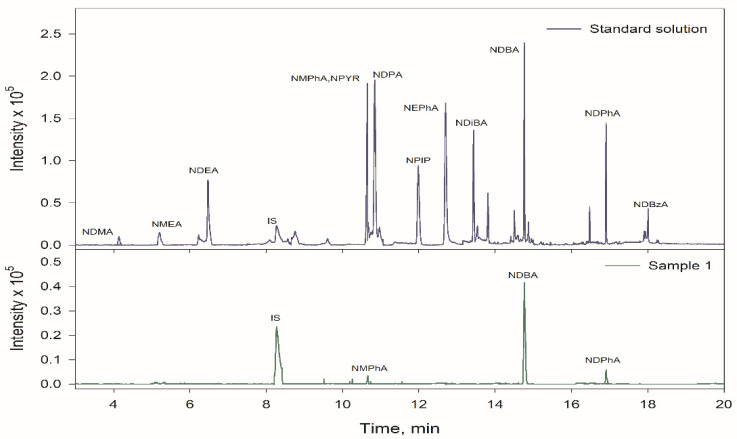
Chromatograms obtained for a standard solution mixture at 50 ng g^−1^ for all the compounds and unfortified sample 1 using the optimized method.

Figure 2 shows the chromatogram in SIM mode for a standard solution at 50 ng g^−1^ concentration for all NAs submitted to the proposed procedure. The absence of interfering peaks at the retention times of the NAs corroborates the selectivity of the method. Figure 2 also shows an extracted ion chromatogram for the unfortified sample 1 at room temperature. Identification of the compounds was performed by comparison of their retention times and mass spectra for standard solutions and fortified samples.

The trueness of the method was evaluated by recovery studies because no certified reference materials were available. For this, three samples (1, 5 and 7) fortified at two concentrations (10 and 50 ng g^−1^) were prepared in duplicate. The results show that the recoveries were 86.8–105.9% (*n* = 144) for the lower level and 84.1–111.2% (*n* = 144) for the higher level for all types of samples.

## 4. Conclusions

An evaluation of the effect of elastic rubber nettings on the levels of twelve N-nitrosamines in meat products established that there is no relationship between the elastic rubber nettings used in the manufacturing process and the NAs the products contain, since there were no differences between the levels found in the products made with several plastics or thread in the presence of additives. The procedure, based on the combination of two miniaturized analytical techniques (UAE and DLLME with GC–MS), is an excellent and quick procedure for the quality control of NAs in meat samples. In addition, the temperature study revealed that there was only a very slight variation in the concentrations of the NAs at the different temperatures to which the samples were submitted.

## Figures and Tables

**Table 1:** Chromatographic and detection parameters for analysis of NAs.

Compound	t_R_, min	Target Ion, *m*/*z*	Qualifier Ion, *m*/*z*
NDMA	4.13	74	31, 42, 44
NEMA	5.23	88	42, 44, 56
NDEA	6.48	102	42, 44, 57
Octanone (IS)	8.27	58	43
NMPhA	10.65	106	44, 77, 107
NPYR	10.67	100	41, 42, 68
NDPA	10.85	70	42, 43, 130
NPIP	11.99	114	42, 44, 55
NEPhA	12.70	106	77, 121
NDiBA	13.43	84	43, 57
NDBA	14.76	84	41, 57, 116
NDPhA	16.91	169	167, 168
NDBzA	18.01	91	44, 88

IS, internal standard; NAs, nitrosamines; NDMA, N-nitrosodimethylamine; NEMA, N-nitrosoethylmethylamine; NDEA, N-nitrosodiethylamine; NMPhA, N-nitroso-methylphenylamine; NPYR, 1-nitrosopyrrolidine; NDPA, N-nitrosodi-n-propylamine; NPIP, 1-nitrosopiperidine; NEPhA, N-nitroso-ethylphenylamine; NDiBA, N-nitrosodiisobutylamine; NDBA, N-nitrosodibutylamine; NDPhA, N-nitrosodiphenylamine; NDBzA, N-nitrosodibenzylamine.

**Table 2:** Analytical characteristics of the DLLME-GC–MS method for NAs determination.

Compounds	Linearity, ng g^−1^	QL, ng g^−1^	DL, ng g^−1^	RSD ^a^, %	RSD ^b^, %
NDMA	5–1000	4.4	1.3	4.2 (5.5)	5.2 (6.9)
NEMA	5–1000	2.2	0.67	4.1 (5.2)	4.3 (5.4)
NDEA	5–1000	3.9	1.4	5.7 (7.5)	8.9 (10)
NMPhA	1–1000	0.7	0.20	4.8 (6.3)	5.1 (6.5)
NPYR	5–1000	1.5	0.5	4.0 (5.2)	4.6 (5.9)
NDPA	1–1000	0.9	0.3	6.9 (10)	9.2 (11)
NPIP	2.5–1000	1.4	0.42	6.2 (8.9)	9.7 (12)
NEPhA	1–1000	0.8	0.24	5.7 (7.6)	6.0 (7.9)
NDiBA	2.5–1000	1.6	0.48	5.9 (7.7)	6.2 (8)
NDBA	1–1000	0.5	0.15	4.2 (5.2)	8.8 (9.7)
NDPhA	2.5–1000	1.2	0.4	2.5 (3.3)	5.0 (6.4)
NDBzA	5–1000	4.6	1.4	3.8 (4.9)	4.0 (5.2)

^a^ Intraday analysis (*n* = 9). ^b^ Inter-day analysis (*n* = 21). Values in brackets refer to the analysis without IS. DL, detection limit; DLLME, dispersive liquid-liquid microextraction; GC, gas chromatography; MS, mass spectrometry; QL, quantification limit; RSD, relative standard deviation.

**Table 3:** Comparison of the proposed method with others previously published for NA quantification in meat by GC–MS.

NAs	Sample Treatment			DLs (ng g^−1^)	NA Levels, ng g^−1^	Ref.
	Technique	Sample Mass, g	Time ^a^, min			
9 (NEMA, NDMA, NDEA, NPYR, NMOR, NDPA, NPIP, NDBA NDPhA)	MAE-DLLME	0.25	70/5	0.12–0.56	ND-5.7	[2]
7 (NDMA, NDEA, NDBA, NMEA, NPIP, NDPhA, NPYR)	MAE-DLLME	1.5	25/10	0.11–0.48	ND-8.6	[5]
9 (NDMA, NDEA, NEMA, NDPA, NMOR, NPYR, NPIP, NDBA, NDPhA)	HS-SPME	1	55	<3.6 56 for NDMA	ND-5.0	[22]
7 (NDMA, NPYR, NDPA, NPIP, NDBA NMEA, NDEA)	MAE-D-µ-SPE	5	10/35	0.01–0.12	ND-3.2	[24]
9 (NDMA, NDEA, NDPA, NDBA, NPIP, NPYR, NMOR, NDPhA, NMEA)	HS-SPME	2	35	7.2–16	ND-10	[36]
7 (NMEA, NDBA, NDPA, NDEA, NDMA, NPIP, NPYR)	MAE-DLLME	1	65/6	0.1–0.5	<0.1–4.8	[41]
8 (NDMA, NEMA, NDEA, NPIP, NMOR, NDPA, NPYR, NDBA)	SLE-SPE	10	70/60	0.05–0.10	0.1–22.1	[3]
12 (NDMA, NEMA, NDEA, NPYR, NDPA, NPIP, NDBA, NDPhA, NMPhA, NEPhA, NDiBA, NDBzA)	UAE-DLLME	1	10/3	0.15–11	ND-40	This method

^a^ Extraction step/preconcentration step. HS, headspace; MAE, microwave-assisted extraction; SLE, solid-liquid extraction; SPE, solid-phase extraction; D-µ-SPE, dispersive micro SPE; ND, not detected; SPME, solid-phase microextraction; UAE, ultrasound-assisted extraction.

**Table 4:** Average concentration ^a^ found (ng g^−1^) in cooked ham samples.

Sample Number	Elastic Rubber Netting ^b^	Meat	Additive	Concentration Found, ng g^−1^				
				NDMA	NDBA	NDPhA	NMPhA	NDBzA
1	No	Pig	NATPRE	ND	27 ± 2	2.6 ± 0.1	1.8 ± 0.3	ND
2	Prolan V-22	Pig	No	ND	33 ± 3	2.5 ± 0.2	2.3 ± 0.9	15 ± 2
3	Prolan V-22	Pig	NATPRE	ND	23 ± 1	3.7 ± 1	1.6 ± 0.2	ND
4	Prolan V-22	Pig	20 mg kg^−1^ nitrite	8 ± 4	23 ± 6	3.4 ± 0.1	1.4 ± 0.2	28 ± 3
5	Prolan V-22	Chicken	NATPRE	14 ± 9	39 ± 2	2.5 ± 0.3	1.4 ± 0.2	ND
6	Prolan M-0 V-6-4 HGB-3 XL	Pig	NATPRE	11 ± 1	22 ± 3	1.9 ± 0.1	1.5 ± 0.3	ND
7	Prolan V-66 HGB-45 REG	Pig	150 mg kg^−1^ nitrite	ND	40 ± 9	2.7 ± 0.5	1.6 ± 0.3	ND

^a^ Two different areas (inner and outer meat), each in triplicate; ^b^ Polyamide/polyolefin composition with different permeability to oxygen and water vapour.

## References

[1] Lu S., Wu D., Li G., Lv Z., Gong P., Xia L., Sun Z., Chen G., Chen X., You J. (2017). Facile and sensitive determination of N-nitrosamines in food samples by high-performance liquid chromatography via combining fluorescent labeling with dispersive liquid-liquid microextraction. Food Chem..

[2] Campillo N., Viñas P., Martínez-Castillo N., Hernández-Córdoba M. (2011). Determination of volatile nitrosamines in meat products by microwave-assisted extraction and dispersive liquid-liquid microextraction coupled to gas chromatography-mass spectrometry. J. Chromatogr. A.

[3] Wang Z., Zhai M., Xia X., Yang M., Han T., Huang M. (2018). A simple method for monitoring eight N-nitrosamines in beef jerkys by gas chromatography-tandem mass spectrometry with one-step treatment coupled to active carbon solid-phase extraction. Food Anal. Methods.

[4] Ozel M.Z., Gogus F., Yagci S., Hamilton J.F., Lewis A.C. (2010). Determination of volatile nitrosamines in various meat products using comprehensive gas chromatography-nitrogen chemiluminescence detection. Food Chem. Toxicol..

[5] Ramezani H., Hosseini H., Kamankesh M., Ghasemzadeh-Mohammadi V., Mohammadi A. (2014). Rapid determination of nitrosamines in sausage and salami using microwave-assisted extraction and dispersive liquid–liquid microextraction followed by gas chromatography–mass spectrometry. Eur. Food Res. Technol..

[6] Sen N.P. (1988). Migration and Formation of N -Nitrosamines from Food Contact Materials. Food and Packaging Interactions.

[7] Fiddler W., Pensabene J.W., Gates R.A., Adam R. (1998). Nitrosamine formation in processed hams as related to reformulated elastic rubber netting. J. Food Sci..

[8] Sen N.P., Baddoo P.A., Seaman S.W. (1993). Nitrosamines in cured pork products packaged in elastic rubber nettings: An update. Food Chem..

[9] Fiddler W., Pensabene J.W., Gates R.A., Custer C., Yoffe A., Phillipo T. (1997). N-Nitrosodibenzylamine in boneless hams processed in elastic rubber nettings. J. AOAC Int..

[10] Pensabene J.W., Fiddler W., Gates R.A. (1995). Nitrosamine formation and penetration in hams processed in elastic rubber nettings: N-nitrosodibutylamine and N-nitrosodibenzylamine. J. Agric. Food Chem..

[11] Pensabene J.W., Fiddler W., Gates R.A. (1992). Solid-Phase extraction method for volatile N-nitrosamines in hams processed with elastic rubber netting. J. AOAC Int..

[12] Sen N.P., Seaman S.W., Kushwaha S.C. (1987). Improved method for determination of volatile nitrosamines in baby bottle rubber nipples and pacifiers. J. Assoc. Off. Anal. Chem..

[13] Bouma K., Schothorst R.C. (2003). Identification of extractable substances from rubber nettings used to package meat products. Food Addit. Contam..

[14] Petersen A. (1993). N-Nitrosodibutylamine and other Volatile Nitrosamines in Cured Meat Packaged in Rubber Nettings. J. Food Sci..

[15] Helmick J.S., Fiddler W. (1994). Thermal decomposition of the rubber vulcanization agent, zinc dibenzyldithiocarbamete, and its potential role in nitrosamine formation in hams processed in elastic nettings. J. Agric. Food Chem..

[16] (1995). European Parliament and Council of the European Union European Parliament and Council Directive No 95/2/EC of 20 February 1995 on food additives other than colours and sweeteners. Off. J. Eur. Union.

[17] Crews C. (2010). The determination of N- nitrosamines in food. Qual. Assur. Saf. Crop. Foods.

[18] Herrmann S.S., Duedahl-Olesen L., Granby K. (2015). Occurrence of volatile and non-volatile N-nitrosamines in processed meat products and the role of heat treatment. Food Control.

[19] Fan C.C., Lin T.F. (2018). N-nitrosamines in drinking water and beer: Detection and risk assessment. Chemosphere.

[20] Lona-Ramirez F.J., Gonzalez-Alatorre G., Rico-Ramírez V., Perez-Perez M.C.I., Castrejón-González E.O. (2016). Gas chromatography/mass spectrometry for the determination of nitrosamines in red wine. Food Chem..

[21] Cintya H., Silalahi J., De Lux Putra E., Siburian R. (2019). Analysis of nitrosamines in processed meat products in Medan city by liquid chromatography-mass spectrometry. Open Access Maced. J. Med. Sci..

[22] Roasa J., Liu H., Shao S. (2019). An optimised HS-SPME-GC-MS method for the detection of volatile nitrosamines in meat samples. Food Addit. Contam. Part A Chem. Anal. Control. Expo. Risk Assess..

[23] Filho P.J.S., Rios A., Valcárcel M., Melecchi M.I.S., Caramão E.B. (2007). Method of determination of nitrosamines in sausages by CO2 supercritical fluid extraction (SFE) and micellar electrokinetic chromatography (MEKC). J. Agric. Food Chem..

[24] Huang M.C., Chen H.C., Fu S.C., Ding W.H. (2013). Determination of volatile N-nitrosamines in meat products by microwave-assisted extraction coupled with dispersive micro solid-phase extraction and gas chromatography-Chemical ionisation mass spectrometry. Food Chem..

[25] Soares F.A., Chiapetta S.C., Pacheco W.F. (2017). Development of an analytical method for the determination of N-nitrosamines in tobacco by GC-NCD after solid phase extraction. Anal. Methods.

[26] Jurado-Sánchez B., Ballesteros E., Gallego M. (2011). Gas chromatographic determination of N-nitrosamines, aromatic amines, and melamine in milk and dairy products using an automatic solid-phase extraction system. J. Agric. Food Chem..

[27] Zhang Q., Jin L., Zhang F., Yao K., Ren Y., Zhang J., Zhang Q., He Q., Wan Y., Chi Y. (2019). Analysis of 7 volatile N-nitrosamines in Chinese Sichuan salted vegetables by gas chromatography-tandem mass spectrometry coupled to modified QuEchERS extraction. Food Control.

[28] Qiu Y., Chen J.H., Yu W., Wang P., Rong M., Deng H. (2017). Contamination of Chinese salted fish with volatile N-nitrosamines as determined by QuEChERS and gas chromatography–tandem mass spectrometry. Food Chem..

[29] Seo J.-E., Park J.-E., Lee J.-Y., Kwon H. (2016). Determination of Seven N-nitrosamines in Agricultural Food Matrices Using GC-PCI-MS/MS. Food Anal. Methods.

[30] Wang X., Gao Y., Xu X., Zhao J., Song G., Hu Y. (2011). Derivatization method for determination of nitrosamines by GC-MS. Chromatographia.

[31] Jurado-Sánchez B., Ballesteros E., Gallego M. (2007). Gas chromatographic determination of N-nitrosamines in beverages following automatic solid-phase extraction. J. Agric. Food Chem..

[32] Kühne F., Kappenstein O., Straβgütl S., Weese F., Weyer J., Pfaff K., Luch A. (2018). N-nitrosamines migrating from food contact materials into food simulants: Analysis and quantification by means of HPLC-APCI-MS/MS. Food Addit. Contam. Part A Chem. Anal. Control. Expo. Risk Assess..

[33] Li W., Chen N., Zhao Y., Guo W., Muhammd N., Zhu Y., Huang Z. (2018). Online coupling of tandem liquid-phase extraction with HPLC-UV for the determination of trace: N -nitrosamines in food products. Anal. Methods.

[34] Bian Y., Zhang Y., Zhou Y., Li G.-H., Feng X.-S. (2020). Progress in the pretreatment and analysis of N-nitrosamines: An update since 2010. Crit. Rev. Food Sci. Nutr..

[35] Aragón M., Marcé R.M., Borrull F. (2013). Determination of N-nitrosamines and nicotine in air particulate matter samples by pressurised liquid extraction and gas chromatography-ion trap tandem mass spectrometry. Talanta.

[36] Sun C., Wang R., Wang T., Li Q. (2020). Primary evaluation of nine volatile N-nitrosamines in raw red meat from Tianjin, China, by HS-SPME-GC–MS. Food Chem..

[37] Ventanas S., Ruiz J. (2006). On-site analysis of volatile nitrosamines in food model systems by solid-phase microextraction coupled to a direct extraction device. Talanta.

[38] Lobato V., Rath S., Reyes F.G.R. (2006). Effect of processing on the degradation of ivermectin in milk and milk products. Toxicol. Lett..

[39] Andrade R., Reyes F.G.R., Rath S. (2005). A method for the determination of volatile N-nitrosamines in food by HS-SPME-GC-TEA. Food Chem..

[40] Sen N.P., Seaman S.W., Page B.D. (1997). Rapid semi-quantitative estimation of N-nitrosodibutylamine and N-nitrosodibenzylamine in smoked hams by solid-phase microextraction followed by gas chromatography-thermal energy analysis. J. Chromatogr. A.

[41] Amelin V.G., Lavrukhin D.K. (2016). Combination of microwave heating extraction and dispersive liquid-liquid microextraction for the determination of nitrosoamines in foods using gas-liquid chromatography with a mass-spectrometric detector. J. Anal. Chem..

[42] Giménez-Campillo C., Pastor-Belda M., Campillo N., Hernández-Córdoba M., Viñas P. (2020). Development of a new methodology for the determination of N-Nitrosamines impurities in ranitidine pharmaceuticals using microextraction and gas chromatography-mass spectrometry. Talanta.

[43] (2002). Commission Decision 2002/657/EC Commission Decision 2002/657/EC of 12 August 2002 implementing Council Directive 96/23/EC concerning the performance of analytical methods and the interpretation of results. Off. J. Eur. Union.

[44] Goss L.C., Monthey S., Issel H.M. (2006). Review and the latest update of n-nitrosamines in the rubber industry; The regulated, the potentially regulated, and compounding to eliminate nitrosamine formation. Rubber Chem. Technol..

